# The correlated influence of semantic types, L1 translation equivalents and language proficiency on EFL learners' production of polysemous words

**DOI:** 10.3389/fpsyg.2023.1162008

**Published:** 2023-12-19

**Authors:** Chunhong Qu

**Affiliations:** Department of English Education, College of Foreign Languages, Capital Normal University, Beijing, China

**Keywords:** polysemous words, core meanings, dominant L1 translation equivalents, language proficiency, correlated influence

## Abstract

The current research investigated the influencing factors of the full mastery of L2 (English) polysemous words with a Chinese-English translation test. The concepts of “meaning” and “L2 equivalent” were strictly distinguished when designing the test. The manipulation of variables came to a mixed design of 2 (semantic types: core meaning, periphery meaning) × 2 (types of L1 translation equivalents: dominant L1 translation equivalent, non-dominant L1 translation equivalent) × 2 (language proficiency: low proficiency, high proficiency). The results showed that: (1) The semantic types and L1 translation equivalents had significant influence on the production of polysemous words in that the core meanings and the dominant L1 translation equivalent helped the learners access the words quickly and produce them more efficiently; The dominant L1 translation equivalents could facilitate the production of the words presented with their periphery meanings; (2) On the whole, there was no significant difference of the productive competence between the two groups of participants. Comparatively, the high proficiency learners performed better in the production of words presented in their periphery meanings corresponding to non-dominant L1 translation equivalents. And they showed a less degree of dependence on L1 translation equivalents in the process of language production.

## 1 Introduction

Polysemous words, that is, words with multiple meanings, usually contain core meanings and periphery meanings. In most cases, core meanings are acquired prior to the periphery meanings, so L2 learners tend to be more proficient in acquiring the core meanings (Schmitt, 1998), and quite weak in learning the periphery meanings. The core meanings have been put much emphasis in the previous researches, which suggested that the core meaning is a key factor in helping learners acquire periphery meanings (Morimoto and Loewen, 2007; Mark, 2016; Abdul and Alnamer, 2017). However, from the perspective of classroom second language teaching and learning, the function of the core meanings is very likely to be overestimated. One of the reasons is that, if the learners are very familiar with the core meaning of a polysemous word, they tend to stick to the meaning they know (Laufer, 1997) and are reluctant to abandon it even when it is used in a different context with periphery meanings, which can lead to confusion and misunderstanding in both receptive and productive communications (Abdul and Alnamer, 2017). Thus it is a tough task for learners to get full mastery of a polysemous word with both core meanings and periphery meanings. And more ways of acquiring periphery meanings actively should be explored instead of leaving them to chance encounters (Parent, 2009). Another important reason is that in most of the previous studies, the “meaning” is roughly regarded the same as the “L1 translation equivalent,” that is, the “meaning” of an L2 word is usually illustrated by its L1 translation equivalent for the sake of concision or convenience. For example, one of the meanings of *term* is “a word or expression with a particular meaning, especially one that is used for a specific subject or type of language.” But to most Chinese learners, the “meaning” of *term* is *shuyu*, which is the Chinese equivalent of the target word. Similarly, the meaning of *bulge* is presented as *bult* in Dutch instead of “a curved mass on the surface of something, usually caused by something under or inside it” (Verspoor and Lowie, 2003; Miao, 2015). The meanings of target words are seldom distinguished from L1 translation equivalents by the researchers in a strict way, thus make it difficult to determine whether it is the core meanings or L1 translation equivalents impacting the acquisition and production of periphery meanings. And the influencing factors of a full mastery of the polysemous words need further exploration.

Scholars have defined “core meaning” in similar ways, suggesting that core meaning is the meaning that coexists in different concepts of words (Caramazza and Grober, 1976; Ruhl, 1989; Foraker and Murphy, 2012), is the most basic literal meaning or logical central usage of words (Verspoor and Lowie, 2003), is the central, primitive, or constant meaning of a word (Hatch and Brown, 2001, p. 47), and is the meaning that does not depend on any context and is well-known to the users (Ma, 2016, p. 14). Gui (2013, p. 51) pointed out that in common historical representation of word meanings, the core meanings are usually those ranked first in the dictionary. Periphery meanings, on the other hand, are the meanings extended by core semantics through cognitive principles such as metaphor, metonymy, conceptualization, and image schemata transformation (Zhang, 2010). They are highly dependent on context and are the meanings that rank after core semantics in the dictionary.

**Figure 1:**
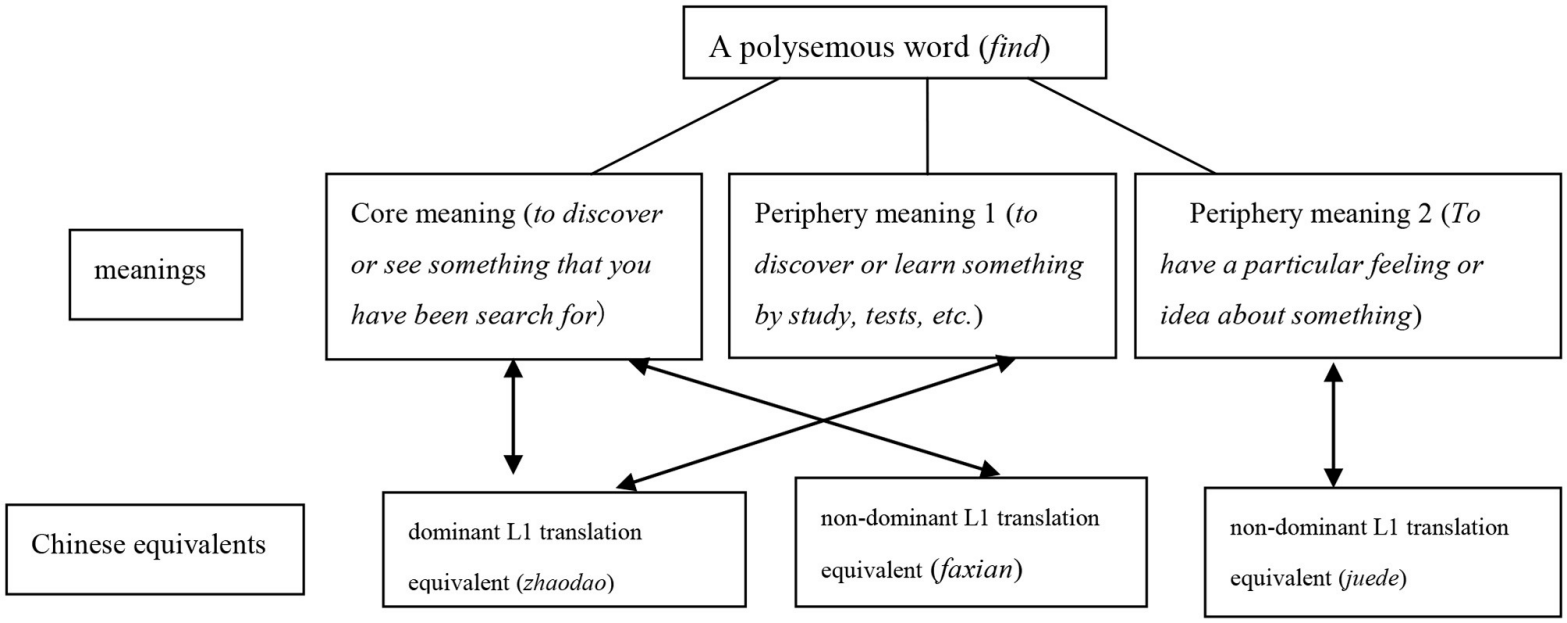
Correspondence between meanings and L1 (Chinese) translation equivalents of the English polysemous word *find*.^a^^a^The definitions of “meanings” and “Chinese equivalents” referred to *Longman's Contemporary Advanced English Dictionary* (2004).

L1 translation equivalents are words in L1 that usually have the same core meanings as L2 words, regardless of whether they have the same periphery meanings (Öztürk, 2018). They include words or expressions overlapping with meanings of L2 words. As shown in Figure 1, a polysemous English word can correspond to multiple L1 (Chinese) equivalents with similar meanings, including dominant L1 translation equivalents and non-dominant L1 translation equivalents (Laxén and Lavaur, 2010). Reversely, one L1 translation equivalent can also express different L2 meanings, including the core meanings and periphery meanings.

Thus, several questions need consideration regarding vocabulary acquisition. Firstly, if the word is presented with its core meaning corresponding to both a dominant and non-dominant L1 translation equivalent, will the dominant L1 equivalent facilitate the access and production of the polysemous word? Secondly, if the word is presented with its periphery meaning corresponding to a dominant L1 translation equivalent, will the dominant L1 translation equivalent facilitate the access and production of the periphery meanings as well? Will there be any differences between the acquisition of the two periphery meanings of polysemous words corresponding to a dominant L1 translation equivalent and a non-dominant L1 translation equivalent? Thirdly, will there be any changes in the impacts of core meanings and L1 translation equivalents with the development of L2 learners' language proficiency? Currently, sufficient empirical data has not been found on these issues. This research, by distinguishing between “meaning” and “L1 translation equivalent,” attempted to examine how the types of meanings and L1 translation equivalents affected the access and production of polysemous words in a Chinese-English translation test, and whether this influence would change along with the development of L2 learners' language proficiency. By exploring these questions, the research would shed lights on the acquisition of polysemous words, especially the acquisition of periphery meanings of polysemous words.

## 2 Literature review

### 2.1 Previous studies on the influence of semantic types and L1 translation equivalents

#### 2.1.1 The influence of semantic types

Many previous studies have focused on the influence of semantic types on the production and acquisition of polysemous words. Verspoor and Lowie (2003) investigated the functions of core meanings and periphery meanings through three vocabulary tests: a test of guessing meaning from context, a short-term memory vocabulary test and a long term vocabulary test. They found that the core meanings were quite helpful for learners to acquire the periphery meanings, and comparatively, providing core meanings could ensure participants learn more words in their long term memory than providing periphery meanings did.

The function of core meanings for recognizing periphery meanings have also been found in other studies. Mark (2016) explored the influencing factors of the acquisition of polysemous words with an acceptability judgment test, finding that even though some distracters in the experiment were not native-like expressions, the participants tend to accept their usage as long as they were close to the core meanings. The results of Wei and Lou (2015) showed that providing core meaning cues can help learners guess the periphery meanings of unfamiliar polysemous words, but only providing them with periphery meanings had little effect in helping them guess the extended meanings of the same words. Abdul and Alnamer (2017) tested the awareness of Arabic learners toward polysemous words with an English-Arabic translation task of three words: *open, run* and *make*. The results suggested that learners could get the core and basic meaning of the polysemous word according to the context, and encounter some difficulties when guessing the extended meanings in some particular contexts. The empirical results of Morimoto and Loewen (2007) and Mitsugi (2017) on teaching methods of polysemous words showed that the pedagogical method of directly teaching core meanings based on schemata was an effective way to learn polysemous words. Mo and Sun (2004), and Zhang (2010) supported that providing core meanings of polysemous words ensured the Chinese learners remember more English words in their long-term memory.

#### 2.1.2 The influence of L1 translation equivalents

Translating words into their native language helps students quickly memorize vocabulary and the L1 translation equivalents have certain features that can be used by the teacher to the learners' advantage (Nation, 1990, p. 62). The L1 translation equivalent is commonly used in classroom vocabulary teaching to define the meaning of a word; however, the L1 equivalent is not always the same as the vocabulary meaning. If the same concept corresponds to multiple L1 translation equivalents, then the one at the top of the lexical entry in the dictionary is usually regarded as the dominant L1 translation equivalent. For example, in *Longman's Contemporary Advanced English Dictionary* (2004), the core meaning of the word *express* is “to tell people what you are feeling or thinking by using words,” and the L1 (Chinese) equivalents are “*biaoshu*” and “*chenshu*.” Among them, “*biaoshu*” presupposes the dominant L1 translation equivalent.

Wang and Zhang (2013) investigated the proficient Chinese-English bilinguals' recognition process and relative factors of polysemous words. The results suggested that when words were presented with their dominant L1 translation equivalent, the recognition process was faster than presented in their non-dominant translation equivalent. However, as mentioned earlier, most previous studies did not strictly distinguish between “meaning” and “L1 translation equivalent.” As a result, it is difficult to determine whether it is the type of lexical meanings or L1 translation equivalents that affect the production of the polysemous words.

Researches about bilingual mental lexicon found that learners tend to rely on the L1 lexical system in learning new words in a second language, because the meanings of an L2 word can be understood through their L1 translation (Jiang, 2000, 2002). The learners access the meanings of L2 words with the help of their L1 translation equivalents (Potter et al., 1984; Kroll and Stewart, 1994). Öztürk (2018) investigated the effects of sense type and L1 translations on the acquisition of noun polysemous words with an acceptability judgment test. The sense types included the core senses and two extended senses: metonymical and metaphorical senses; the corresponding L1 translation equivalents included four categories: parallel senses, L2-only senses, L1-only senses, and nonce senses. The results of the study indicated that both of two variables were effective. The core senses were acquired better than the corresponding extended senses. The parallel senses in the L1 facilitated learners' performance in the case of metonymical senses.

### 2.2 Previous studies on the influence of language proficiency

Language proficiency is another factor impacting the acquisition of polysemous meanings. In early studies, Schmitt (1998) tracked longitudinally the development of three English learners' knowledge of 11 polysemous words and concluded that advanced non-native speakers might have mastery over only a rather limited number of the possible meanings of a word, even if they were proficient enough to study in British universities. Laufer (1997) investigated the acquisition of lexical knowledge through diachronic surveys and comparisons of learners of different grades. She found that the knowledge of learners' productive vocabulary did not grow significantly with the development of their language proficiency.

The studies of Wu and Chen (2000) and Tan (2006) suggested that during the low proficiency stage, the access and productive capacity of the learners were significantly improved, but at the advanced stage, there would be a fossilization state or plateau phenomenon, in which the tendency of the improvement slowed down and even stopped. Zhang's (2010) investigation from the perspective of psycholinguistics showed that for low proficiency learners, the core meanings of polysemous words held priority in vocabulary access and production, and with the improvement of language proficiency, the representation of periphery meanings gradually increased. In the study of Wei and Lou (2015), undergraduates and postgraduates showed similar acquisition effects in core meaning, but the acquisition of periphery meanings was different. The scores of the postgraduates are higher.

In the previous studies, the analysis was made about the influence of semantic types, L1 translation equivalents and language proficiency. However, there have been few studies distinguishing between “meaning” and “L1 translation equivalent,” thus it is difficult to determine the functions of semantic types and L1 translation equivalents in lexical production. Moreover, the correlated influence of those factors is seldom tackled in the previous studies, especially the influence of semantic types and L1 translation equivalents on the learners' of different language proficiency also need further discussion. Therefore, in order to have a clearer view about the influencing factors and their relationship on the production of L2 polysemous words of high and low proficiency learners, a Chinese-English translation test was designed.

## 3 Research design

### 3.1 Research questions

The research explored the impact of semantic types, L1 translation equivalents, and language proficiency on the production of polysemous words through a Chinese-English translation test. One of the independent variables was semantic types with two levels (core meaning and periphery meaning). The second independent variable was L1 translation equivalents, and it was operationalized into two levels: dominant L1 translation equivalent, and non-dominant L1 translation equivalent. The third independent variable was participants' language proficiency, which also had two levels (high proficiency and low proficiency). The dependent variable was the score of Chinese-English translation tests of polysemous words. Therefore, the research adopted a 2 × 2 × 2 mixed design. Specifically, it addressed the following two research questions:

(1) How do semantic types and L1 translation equivalents influence the production of English polysemous words in the Chinese-English translation test?
(2) Are there any differences between the high and low proficiency learners in the production of polysemous words? If so, what are the differences?

Three hypotheses were generated accordingly:

Hypothesis 1: Semantic types and L1 translation equivalents have a significant influence on the production of polysemous words. That is, if the target word is presented with its core meaning or dominant L1 translation equivalent in the source Chinese context, the learners are more likely to produce the polysemous word accurately. According to the results of previous studies (Nation, 1990, p. 62; Wang and Zhang, 2013; Mark, 2016), there may be a significant interaction between semantic types and L1 translation equivalents on the production of the polysemous words. The dominant L1 translation equivalent may have a facilitate effect on the production of polysemous words presented with their periphery meanings.
Hypothesis 2: According to the research of Zhang (2010) and Wei and Lou (2015), language proficiency can significantly influence the production of polysemous words. That is, the learners of high proficiency may have a better performance in the production of the polysemous words.
Hypothesis 3: There is a significant interaction between semantic types, L1 translation equivalents and language proficiency on the production of polysemous words. Both high proficiency and low proficiency learners may produce words presented with their periphery meanings more accurately with the facilitation of the dominant L1 translation equivalents, but the high proficiency learners may have a better mastery of the words presented with their periphery meanings corresponding to non-dominant L1 translation equivalents than low proficiency learners do.

### 3.2 Participants

A total of 48 freshmen students and 45 graduates were recruited from a teacher training college in mainland China, and the former group was assumed to be at a low-proficiency level in English and the latter at a high-proficiency level. In order to validate the assumption, the author conducted a pretest of language proficiency with *Oxford Quick Placement Test* (Version 2) in the two groups. An independent-samples *t* test was run and the results indicated that there was a significant difference in English proficiency between the two groups (*t* = 6.85, *df* = 44, *p* < 0.05). Besides this difference, they also belonged to different age groups: the 45 graduates were 23 to 26 years old (*M* = 23.9, *SD* = 0.90) while the 48 first-year college students were 18 to 20 years old (*M* = 18.56, *SD* = 0.71).

### 3.3 Materials

A random sampling was adopted in this research. Altogether 22 English high-frequency polysemous words were randomly selected from the first 2,000 words of the *English General Service Word List* as target words, including nouns, verbs, and adjectives. Each word was presented with their core meanings and one of their periphery meanings. And each type of meaning corresponds to two kinds of L1 (Chinese) equivalents: a dominant L1 equivalent and a non-dominant L1 equivalent. Thus four categories of variable combinations were formed, namely, “dominant L1 equivalent + core meaning” (DE-CM), “non-dominant L1 equivalent + core meaning” (NDE-CM), “L1 dominant equivalent + periphery meaning” (DE-PM) and “non-dominant L1 equivalent + periphery meaning” (NDE-PM). Accordingly, each word was put into 4 Chinese contexts and altogether 88 Chinese sentences were created to design a Chinese-English translation test. Table 1 below presents the examples of the four variables of the word *find*, their Chinese contexts in the Chinese-English test and the production of the target word in the possible English translations.

**Table 1:** Examples of materials.

**The target word**	**L1 translation equivalents and semantic types**			
	**DE-CM**	**NDE-CM**	**DE-PM**	**NDE-PM**
*find*	*zhaodao* (to discover or see something that you have been searching for.)	*faxian* (to discover or see something that you have been searching for.)	*zhaodao* (to discover or learn something by study, tests, etc.)	*juede* (to have a particular feeling or idea about something)
Sentences in the test	*Haimeiyouren **zhaodao** jiejue zhege wenti de banfa*.	*Wo **faxian** le yiliang henhao de ershouche*.	*Women jiujing nengbuneng **zhaodao** zhiliao zhezhongbing de yao?*	*Wo renshi de henduo nvxing dou **juede** ta henyou meili*.
English translation of the sentences	No one has *found* a solution to this problem.	I *found* a nice second-hand car.	Will we ever *find* a cure for the disease?	Lots of women I know *find* him attractive.

DE-CM, the dominant L1 equivalent corresponding to the core meaning of the word; NDE-CM, the non-dominant L1 equivalent corresponding to the core meaning of the word; DE-PM, the dominant L1 equivalent corresponding to the periphery meaning of the word; NDE-PM, the non-dominant L1 equivalent corresponding to the periphery meaning of the word.

The core meanings and periphery meanings of the polysemous words were decided by referring to *Longman Dictionary of Contemporary English* (2004), in which the first meaning of each entry is the most basic meaning that was used most frequently in daily life (Pxxi, Guide to the dictionary), and the other meanings are periphery meanings. The L1 translation equivalents, on the other hand, were decided according to both the lexical entry in the same dictionary and the result of a pretest. According to the Chinese translations provided in the dictionary, the first L1 translation equivalent of the core meanings was regarded as the dominant L1 translation equivalent, and the others as the non-dominant L1 translation equivalents. In order to determine whether the L1 dominant equivalent was the preferred translation word in L2 learners' mental lexicon, a pretest of English-Chinese word translation was designed as some researchers did in their previous studies (Wang and Zhang, 2013; Qu, 2022). A group of 20 English learners who would not participate in the experiment took part in the test. They were asked to translate the 22 target English words into their Chinese equivalents. They should come up with the Chinese words as quickly as possible to ensure that the Chinese equivalents they produced were the most salient ones in their mental lexicon. If a Chinese equivalent was chosen by over 80% of the participants as the first translation equivalent of the English word, and was also identical with the first Chinese translation in the dictionary, it was determined to be the dominant L1 equivalent.

As shown in Table 1, the dominant L1 translation equivalent could present both the core meaning and one of the periphery meanings of the target words. The non-dominant L1 translation equivalents were chosen randomly from the other Chinese translations of the core meanings and one of the periphery meanings of the lexical entry in the dictionary.

### 3.4 Data collection

The participants were provided with a set of 88 Chinese sentences in the test and were required to translate each sentence into English. They completed the test online. In the test, all sentences appeared at random; no special instructions about the target words were given; dictionaries or any other reference materials were not allowed. Since the sentences were simple-structured short sentences composed of simple words, it just took the participants about 100 minutes to complete the test.

The results of the test were carefully examined. If the target word was successfully produced in the English translation, 1 point was awarded. The inflections of the target words were also accepted. But if a word other than the target word was produced, 0 points were awarded. Altogether, four sets of data were obtained from the high and low proficiency groups. An ANOVA employing SPSS software package was used to demonstrate whether the effects of the semantic types, L1 translation equivalents and language proficiency on the production of polysemous words were statistically significant.

## 4 Results

### 4.1 Influence of the semantic types and L1 translation equivalents on translation results

The descriptive statistics of the translation results are shown in Table 2. The ANOVA statistics are shown in Table 3.

**Table 2:** Descriptive statistics of the production of English polysemous words.

**L1 translation equivalents and semantic types**	**DE-CM**		**NDE-CM**		**DE-PM**		**NDE-PM**	
	***M***	***SD***	***M***	***SD***	***M***	***SD***	***M***	***SD***
High-proficiency group (*n* = 45).	17.49	2.03	12.93	2.05	16.89	2.19	5.38	1.60
Low-proficiency group (*n* = 45).	17.47	1.52	13.47	2.51	17.07	2.23	4.69	1.68

DE-CM, the dominant L1 equivalent corresponding to the core meaning of the word; NDE-CM, the non-dominant L1 equivalent corresponding to the core meaning of the word; DE-PM, the dominant L1 equivalent corresponding to the periphery meaning of the word; NDE-PM, the non-dominant L1 equivalent corresponding to the periphery meaning of the word.

**Table 3:** ANOVA statistics.

		**LP**	**CM**	**L1TE**	**Sum of Squares**	**df**	**Mean Square**	**F**	**Sig**.
LP		Linear term			6.41	1	6.41	0.00	1.00
	Derivation	Linear term			447.75	44	10.18		
CM			Linear term		1,690.00	1	1,690.00	720.19^***^	0.00
	Derivation		Linear term		103.25	44	2.35		
L1TE				Linear term	5,921.11	1	5,921.11	3,851.76^***^	0.00
	Derivation			Linear term	67.64	44	1.54		
LP ^*^ CM		Linear term	Linear term		5.88	1	5.88	3.57	0.06
	Derivation	Linear term	Linear term		72.37	44	1.65		
LP ^*^ L1TE		Linear term		Linear term	0.54	1	0.54	0.27	0.60
	Derivation	Linear term		Linear term	88.21	44	2.01		
CM ^*^ L1TE			Linear term	Linear term	1322.50	1	1322.50	663.13^***^	0.00
	Derivation		Linear term	Linear term	87.75	44	1.99		
LP ^*^ CM ^*^ L1TE		Linear term	Linear term	Linear term	11.38	1	11.38	3.95	0.05
	Derivation	Linear term	Linear term	Linear term	126.87	44	2.88		

LP, Language proficiency; CM, Core meaning; L1TE, L1 translation equivalent; ^***^p < 0.001.

The results revealed that L1 translation equivalents significantly influenced the production of polysemous words [*F*_(1, 44)_ = 3,851.76, *p* < 0.05], and the score of words presented with their dominant L1 translation equivalents was significantly higher than that of non-dominant L1 translation equivalents (*MD* = 8.11, *p* < 0.05). The semantic types also significantly influenced the production of the polysemous words [*F*_(1, 44)_ = 720.19, *p* < 0.05]. The score of words presented with their core meanings was significantly higher than that of periphery meanings (*MD* = 4.33, *p* < 0.05). No significant influence of language proficiency was observed (*p* > 0.05). The results showed, as what has been hypothesized, that the types of L1 translation equivalents had a significant influence on the translation scores, and if the dominant L1 translation equivalent of the target word was used in the sentence, the learners tended to produce the word more accurately. Additionally, the semantic types also had a significant influence on scores, such that if the words were presented with their core meanings in the sentences, the accuracy of the target word production would become higher. However, the result about the influence of language proficiency was different from what had been hypothesized about the performances of the two groups.

The semantic types and the types of L1 translation equivalents had a significant correlated influence on the production of the polysemous words [*F*_(1, 44)_ = 663.13, *p* < 0.05]. The scores of the words presented with their core meanings was significantly higher than those with periphery meanings regardless of the types of L1 translation equivalent used in the sentences (*MD* = 0.50, *p* < 0.05; *MD* = 8.17, *p* < 0.05), but the disparity between the scores of NDE-CM and NDE-PM was even greater. The statistics proved that when words presented in their core meanings and periphery meanings both corresponds to their dominant L1 equivalents, the former outperformed the latter with the mean difference of only 0.50; however, when words presented in their core meanings and periphery meanings both corresponds to their non-dominant L1 equivalents, the mean difference became as high as 8.17. The scores of words presented with their dominant L1 translation equivalents was significantly higher than those with non-dominant L1 translation equivalents for both core and periphery meanings (*MD* = 4.28, *p* < 0.05; *MD* = 11.94, *p* < 0.05), and the disparity between the scores of DE-PM and NDE-PM was even greater. Statistics indicated that if the words were presented with their core meanings, the mean difference between dominant L1 translation equivalents and non-dominant L1 equivalents was 4.28; but when the words presented with their periphery meaning, the mean difference between dominant L1 translation equivalent and non-dominant L1 equivalent increased to 11.94. All in all, the scores of the four groups of materials were ranked from highest to lowest as follows: DE-CM > DE-PM > NDE-CM > NDE-PM. The results showed that when the dominant L1 translation equivalents were used in the sentences, no matter the words were presented with their core meanings or periphery meanings, the accuracy of polysemous word production was higher than that with the non-dominant L1 translation equivalents used in the sentences, and even significantly higher than the production score of the words presented with their core meanings corresponding to the non-dominant L1 translation equivalents (*MD* = 3.70, *p* < 0.05), indicating that the dominant L1 translation equivalent played an even greater role in the production of the polysemous words. More importantly, this result indicated that the dominant L1 translation equivalents could facilitate the production of polysemous words presented with their periphery meanings, which demonstrated that Hypothesis 1 was confirmed.

### 4.2 Relationship between the semantic types, L1 translation equivalents, and language proficiency

No significant correlated influence was observed between the types of L1 translation equivalents and the language proficiency (*p* > 0.05), indicating that the tendency and magnitude of the learners' polysemous word production affected by L1 translation equivalents did not change significantly from low proficiency to high proficiency stage.

The correlated influence of semantic types and language proficiency was marginally significant [*F*_(1, 44)_ = 3.57, *p* = 0.06]. For both high and low proficiency groups, the score of the words presented with their core meanings were significantly higher than those with periphery meanings (*MD* = 4.08, *p* < 0.05; *MD* = 4.59, *p* < 0.05). There was no significant difference in translation scores between the high and low proficiency learners (*p* > 0.05) regardless of the words presented with their core meanings of periphery meanings. The results showed that the production of polysemous words of both high and low proficiency learners was affected by the semantic types; however, the magnitude of the influence did not change significantly from the low proficiency to the high proficiency stage.

The triple correlated influence of semantic types, L1 translation equivalents and language proficiency was marginally significant [*F*_(1, 44)_ = 3.95, *p* = 0.05]. The results of simple effect analysis showed that for both high and low proficiency groups, the scores of the words presented with their dominant L1 translation equivalents were significantly higher than those with non-dominant L1 translation equivalents (*MD* = 4.56, *p* < 0.05; *MD* = 11.51, *p* < 0.05; *MD* = 4.00, *p* < 0.05; *MD* = 12.38, *p* < 0.05). If the words were presented with their core meanings, regardless of the types of L1 translation equivalents used, there was no significant difference between the performance of high proficiency and low proficiency groups; if the words were presented with their periphery meanings, marginal significant difference was found between the performance of the two groups of learners only when the non-dominant L1 translation equivalents were used in the sentences (*MD* = 0.69, *p* = 0.07), and the high proficiency group outperformed the low proficiency group. For the high proficiency group, the difference between the words presented with their core meanings and periphery meanings was marginally significant when the dominant L1 translation equivalents were used in the sentence (*MD* = 0.60, *p* = 0.05), and the scores of words presented with core meanings were higher than those with periphery meanings; when the non-dominant L1 translation equivalents were used in the sentence, the score of words presented with their core meanings was also significantly higher than those with their periphery meanings (*MD* = 8.78, *p* < 0.05). The results showed that the types of L1 translation equivalents had a significant influence on the production of polysemous words for both high and low proficiency groups. The significant influence of semantic types was only manifested in the production of PM-NDE, and the high proficiency learners got higher scores. Learners had a stronger dependence on L1 translation equivalents at low proficiency stage, and this dependence tended to weaken in the performance of the high proficiency learners.

## 5 Discussion

### 5.1 Influence of semantic types and L1 translation equivalents

The overall results of the current research suggest that learners' productive competence of polysemous words is related to specific semantic types, and the polysemous words presented with their core meanings are more easily and accurately produced than those with their periphery meanings, indicating that the core meanings of the polysemous words dominate and stand out in the learners' mental lexicon. This conclusion is consistent with previous findings (Verspoor and Lowie, 2003; Mark, 2016; Abdul and Alnamer, 2017; Öztürk, 2018). This view is close to the idea that there is a prototypical central sense of polysemous words (Nunberg, 1995; Dobrić, 2014), and the senses of the polysemous words are structured with conceptual connections between periphery meanings and core meanings. The core meanings are always acquired prior to periphery meanings and facilitate the acquisition of the periphery meanings. Crossley et al. (2010) holds the similar idea that since the periphery meanings are the result of reasonable inference or calculation from the core meanings according to the context and other pragmatic rules, therefore, the learners acquire periphery meanings by extending core meanings. Zhang (2010) explains from the psycholinguistic view that the core meaning of a polysemous word, due to its high frequency of use, usually has a lower threshold value of vocabulary activation, and comparatively speaking, the periphery meanings have a higher threshold value for activation and are not easily activated in language processing.

The types of L1 translation equivalents have a significant influence on the production of lexical meanings. Second language acquisition in a classroom setting is inevitably influenced by the knowledge of the learners' native language. Usually, the same meaning of a polysemous word corresponds to a set of L1 translation equivalents, for example, the core meanings of “clean” correspond to “*dasao*,” “*qingli*,” “*qingjie*” and so on in Chinese. However, in the learners' mental lexicon, different L1 translation equivalents have different degrees of prominence and frequency. Miao (2015) believes that this is related to the order in which polysemous entries are acquired. She investigated different versions of primary and secondary school textbooks and found that the meanings (L1 translation equivalents) presented with highest frequency of the six target words in the study were consistent with the ones that learners acquire in the earliest stage. This can be explained from the view of Jiang (2000) that the first meaning acquired by learners is the first form-meaning reflection established by the learner between the L2 word form and the concept of its L1 translation equivalent so that most learners will access and produce the L2 word with the assistance of L1 translation equivalents in the process of the lexical development. The repeated mapping between the L2 word form and its L1 translation equivalent gradually strengthens the relationship between the two, so this L1 translation equivalent becomes the dominant equivalent. In addition, the dominant L1 translation equivalent shares more nodes with the target word than the non-dominant equivalent in mental lexicon (Laxén and Lavaur, 2010), and learners are more prone to establish a one-to-one matching relationship between the English word form and the dominant L1 translation equivalent (Mo and Sun, 2004). Therefore, in the process of language production in this test, the dominant Chinese equivalents are more likely to activate the English corresponding polysemous words with higher accuracy.

**Figure 2:**
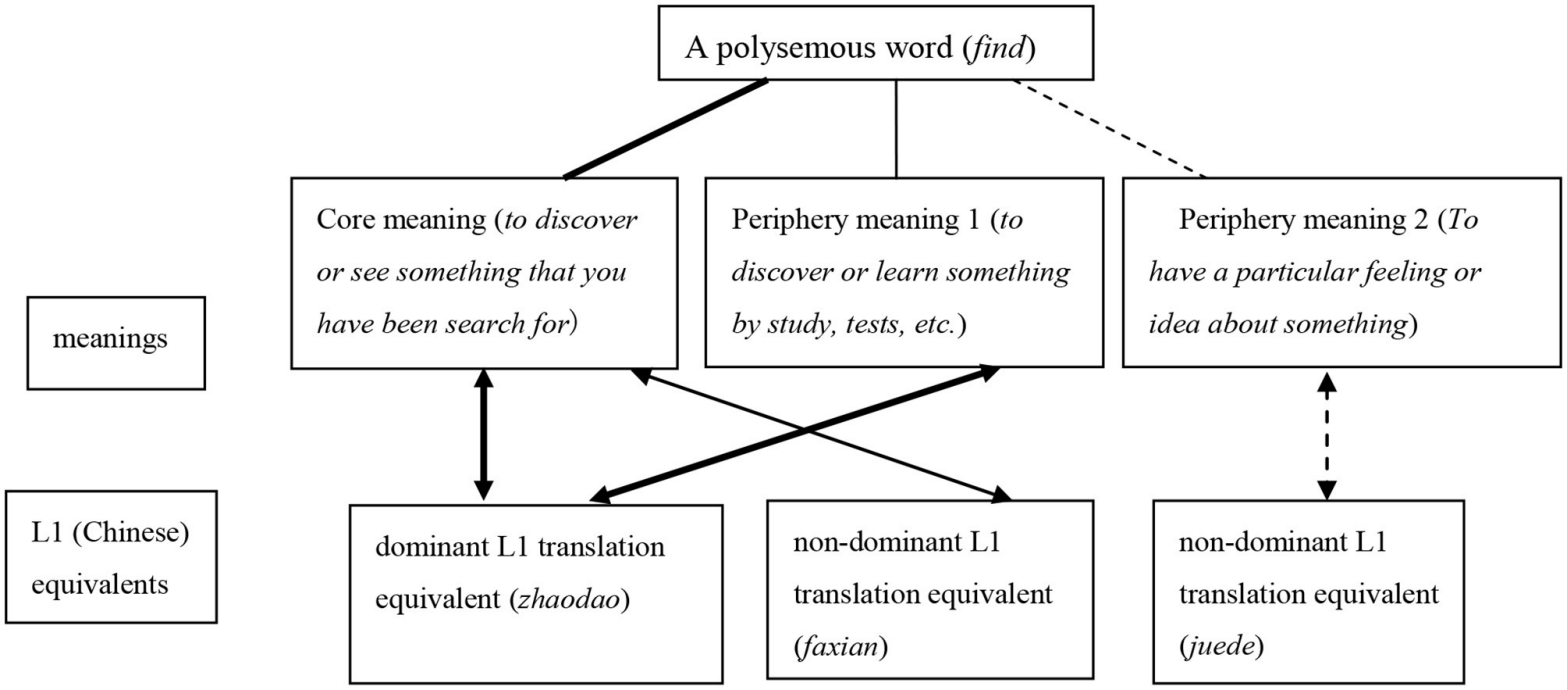
Different degrees of the correspondence between meanings and L1 translation equivalents of a polysemous word.

According to the test results, the semantic types and L1 translation equivalents had significant interaction effects on the production of polysemous words, mainly in that the dominant L1 translation equivalents have a facilitate effect on the production of periphery meanings, which indicated that the dominant L1 translation equivalent can help learners to acquire periphery meanings. This result is somehow inconsistent with the proposal of the prototypical sense theory, which holds that the sequence of the acquisition of the meanings of polysemous words is gradually extended from core meanings to periphery meanings (Nunberg, 1995; Crossley et al., 2010), which ignores the function of the learners' native language in the acquisition of polysemous words. The results of the current research suggest that in the initial stage of second language acquisition, learners tend to obtain L2 semantic representations through the L1 translation equivalents. If the equivalents of the periphery meanings happen to coincide with the dominant L1 translation equivalents, the words presented with their periphery meanings can be activated and accurately produced with the help of the L1 translation equivalents in the language production process. Therefore, from the perspective of the strength of relationship and the function in the lexical acquisition, the lines and arrows in Figure 1 should be revised as is shown in Figure 2. The stronger the lines and arrows are, the easier it is for learners to acquire and produce the word.

Some previous studies have suggested that the acquisition of periphery meanings is one of the difficult points of L2 vocabulary acquisition (Mo and Sun, 2004); however, the results of this study have challenged this presupposition. When the periphery meanings correspond to the dominant L1 translation equivalent, it will facilitate the acquisition and production of periphery meanings. Therefore, it would be more accurate to say that the difficult point of polysemous acquisition is the periphery meanings corresponding to non-dominant L1 translation equivalents. As Figure 2 shows, the words present with their dominant L1 translation equivalents have strongest link with both their core meanings and periphery meanings, which means that the dominant L1 translation equivalents can facilitate the overall acquisition of the polysemous words. However, if the word presents with the core meaning but corresponds with a non-dominant L1 translation equivalent, it may not be learned or produced as accurately as a core meaning corresponding to a dominant translation equivalent. The words presented with their periphery meanings, on the other hand, can sometimes be learned and accessed more quickly with the help of their L1 equivalents. The weakest link is between the periphery meaning and the non-dominant L1 translation equivalent, which illustrates one of the hardest parts that hinders the overall acquisition of polysemous words.

### 5.2 Comparison between high and low proficiency learners

As the current research showed, there was no significant change in L2 learners' production of the polysemous words presented with their core meanings from the low-proficiency stage to the high-proficiency stage. The productive competence of the high proficiency level was significantly higher than the low proficiency group as had been hypothesized, indicating that the productive competence of polysemous words had stagnated in the advanced stage of English learning, which supported the results of previous studies (Schmitt, 1998; Wu and Chen, 2000; Tan, 2006).

Nonetheless, this study also found some differences of learners' lexical productive competence between high and low proficiency groups. The results showed that the high proficiency group scored significantly higher than the low proficiency group for the periphery meanings corresponding to non-dominant L1 translation equivalents. In addition, the effect of the dominant L1 translation equivalents on the semantic types produced by the low proficiency group did not differ significantly but did have a significant impact on the learners at the high proficiency stage. These differences suggested that learners at lower levels are more dependent on the dominant L1 translation equivalents in their language production, tending to complete lexical production directly at the lexical level. With the increase of language proficiency, the connection between the L2 vocabulary and the concept system in learners' mental lexicon has gradually strengthened (Kroll and Stewart, 1994), and the acquisition of polysemous words has gradually developed from the mechanical correspondence of the vocabulary level to the conceptual level. Learners began to build a semantic network of L2 vocabulary and the acquisition and production of L2 words no longer relied solely on L1 translation equivalents.

In the current research, among the four sets of data, NDE-PM scored the lowest, and the average scores were only 5.38 for the high proficiency group and 4.69 for the low proficiency group, which meant that the accuracy rates of the production were only about 24 and 21% for each group. This result highlights the learners' tendency to mechanically map L2 lexical forms to L1 translation equivalents at the lexical level, especially at the early stage of vocabulary acquisition. Suppose Word A presented with NDE-PM happens to correspond to the DE-CM of Word B, then the core meanings of Word B will be activated and produced prior to Word A. For example, the word *find* can be presented with its periphery meaning *to have a particular feeling or idea about something*, and corresponds to a non-dominant L1 translation equivalent of *juede*. And this happens to be identical with the dominant L1 translation equivalent of *feel* (*juede*), and also similar to its core meaning (*feeling to experience a particular feeling or emotion*). Since in the process of language production, the threshold value of core meanings is lower than the periphery meanings, when translating Sentence (1), learners tend to prefer to use *feel* as in sentence (2) rather than *find* as in sentence (3). Though this kind of translation by direct correspondence between the English word and its L1 translation equivalent at lexical level is not an obvious mistranslation, it is slightly rigid in the expression effect, and can influence the accuracy and nativelikeness of the expressions, especially in the high proficiency stage when the higher requirement of learners' language production is expected. And furthermore, as Jiang (2000) has pointed out that not all L2 words have corresponding L1 translations and not all L1 translations have the same degree of semantic overlap with L2 words, the strict one-to-one matching between L2 words and their L1 equivalents may have limited facilitating effect for and production of L2 words, but, in the long run, it will become the one of the main obstacles for the overall acquisition of the meanings of polysemous words.

(1) *Wo renshi de henduo nvxing dou* ***juede*** *ta henyou meili*.
(2) *Lots of women I know feel him attractive*.
(3) *Lots of women I know find him attractive*.

## 6 Conclusion

The current research explored the effects of semantic types, L1 translation equivalents and language proficiency on the production of L2 polysemous words. The results showed that: (1) The core meanings or the dominant L1 translation equivalent facilitated the learners' production of the polysemous words; the dominant L1 translation equivalents also had a facilitating effect on the acquisition and production of the periphery meanings; (2) In general, the productive competence of the high level group was not significantly different from that of the low proficiency group. Compared with the low proficiency group, however, the high proficiency group had developed a stronger competence to produce words presented with their periphery meanings, and the dependence on the dominant L1 translation equivalents was weakened.

From the results, we also found that even for the high proficiency group, the production of words presented with their periphery meanings corresponding to non- dominant L1 translation equivalents was not very accurate. One of the reasons may be that in L2 teaching practice, learners are accustomed to one-to-one matching between L2 lexical form and the concepts of L1 translation equivalents, and have a strong dependence on the conceptual system of the native language. This correspondence was somehow helpful for the acquisition of the words presented with their periphery meanings corresponding to dominant L1 translation equivalents, especially at the low-proficiency stage, but in the long run, it will inevitably exert an inhibited effect on the variety and accuracy of the learners' lexical production. Furthermore, the present study therefore has strong pedagogical implications. More attention needs to be paid to the periphery meanings so that learners can achieve full mastery of the polysemous words, and the productive competence of polysemous words can be improved as a whole. The results of the study, however, could only provide the data of Chinese learners, since no participants of other countries were included in the study. In future studies, a wider range of participants including learners with other languages as their mother tongues need to be employed to further justify the correlated influence of semantic types, L1 translation equivalents and language proficiency on the production of polysemous words.

## Data availability statement

The original contributions presented in the study are included in the article/supplementary material, further inquiries can be directed to the corresponding author.

## Author contributions

CHQ contributed to the conception and design of the study. She organized the database and performed the statistical analysis. She also wrote the first draft of the manuscript, did manuscript revisions and presented the submitted version.
